# When the Appendix Speaks: Colonoscopic Recognition of an Appendiceal Mucocele

**DOI:** 10.7759/cureus.117567

**Published:** 2026-10-06

**Authors:** Caddie Nguyen, Jackelyn Philip, Lucas Youno, Michael Herman

**Affiliations:** 1 Gastroenterology, Lincoln Memorial University-DeBusk College of Osteopathic Medicine, Jacksonville, USA; 2 Gastroenterology, Borland Groover, Jacksonville, USA

**Keywords:** appendiceal mucocele, appendiceal orifice, cecal mass, computed tomography (ct), laparoscopic appendectomy, low-grade appendiceal mucinous neoplasm (lamn), pseudomyxoma peritonei, screening colonoscopy, volcano sign

## Abstract

Appendiceal mucoceles are rare, often asymptomatic lesions caused by mucin accumulation within the appendix and carry a risk of rupture leading to pseudomyxoma peritonei. We report a case of a 60-year-old man undergoing routine screening colonoscopy in whom a smooth, dome-shaped protrusion of the appendiceal orifice was incidentally identified. No biopsy was performed due to concern for underlying appendiceal pathology. Contrast-enhanced CT revealed a 2.2-cm dilated, fluid-filled appendix consistent with a mucocele. The patient underwent elective laparoscopic appendectomy, and histopathology confirmed a low-grade appendiceal mucinous neoplasm confined to the appendix with negative margins. This case highlights the importance of careful endoscopic evaluation of the appendiceal orifice and timely surgical management to prevent complications.

## Introduction

Appendiceal mucocele is a descriptive term for abnormal appendiceal dilatation caused by intraluminal mucin accumulation; it encompasses non-neoplastic obstruction and mucinous neoplasia rather than representing a single histologic diagnosis. It is reported in approximately 0.2%-0.3% of appendectomy specimens [1]. Although appendiceal tumors remain uncommon, their incidence is increasing. A population-based study using U.S. and Canadian cancer registries found that the incidence of malignant appendiceal tumors increased by 232% in the United States and 292% in Canada between 2000 and 2016; this rise occurred across age groups, sexes, stages, and major malignant histologic categories and was not explained by increasing appendectomy rates [2].

Clinical manifestations are often absent or nonspecific, and lesions may be discovered incidentally during imaging, surgery, pathologic examination, or colonoscopy [3,4]. Earlier endoscopic series reported a female predominance, making incidental recognition in an asymptomatic male patient an uncommon demographic presentation [4]. Recognition matters because a smooth appendiceal-orifice bulge may be the only visible clue to an underlying mucinous lesion. When such a lesion is suspected, biopsy or forceful manipulation should be avoided: the overlying mucosa may yield a nondiagnostic sample, whereas disruption can expose or spill mucin and potentially contribute to peritoneal dissemination [5].

## Case presentation

A 60-year-old man presented for a moderate-risk screening colonoscopy. He was asymptomatic and denied abdominal pain, changes in bowel habits, weight loss, nausea, vomiting, or gastrointestinal bleeding. His medical history was notable for hypertension, hyperlipidemia, and gastroesophageal reflux disease. He had no prior abdominal surgeries. Medications included lisinopril, atorvastatin, and omeprazole. Family history was negative for colorectal cancer or inflammatory bowel disease. He was a never-smoker and reported rare alcohol use. Vital signs and physical examination were unremarkable.

Colonoscopy demonstrated a dome-shaped protrusion of the appendiceal orifice with smooth, intact mucosa and a central slit-like opening, concerning for appendiceal mucocele (Figure 1). No ulceration, friability, or mucous extrusion was observed; however, given concern for underlying appendiceal pathology, no biopsy or manipulation of the appendiceal orifice was performed because of the risk of perforation [1]. Although appendiceal mucoceles may demonstrate the classic “volcano sign,” consisting of a circumferential mound with a central appendiceal opening, more subtle findings such as isolated protrusion of the appendiceal orifice may be the only clue [4].

**Figure 1:**
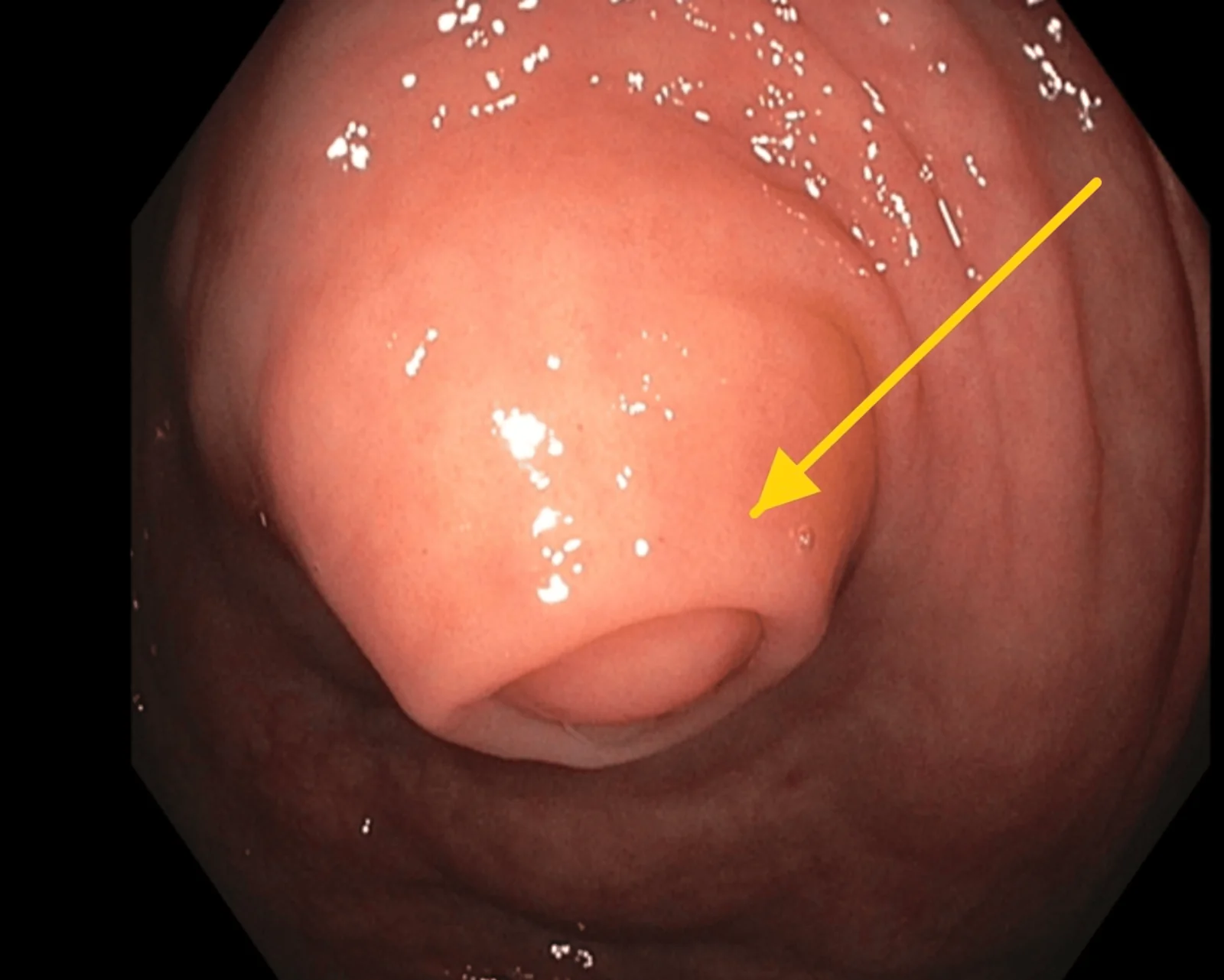
Colonoscopic appearance of an appendiceal-orifice bulge Colonoscopic view demonstrating a smooth, dome-shaped subepithelial protrusion centered on the appendiceal orifice (arrow), with intact overlying mucosa and a central slit-like opening. The appearance raised suspicion for an underlying appendiceal mucocele.

Contrast-enhanced CT abdomen/pelvis subsequently revealed a 2.2-cm dilated, fluid-filled appendix with thin walls and no surrounding inflammatory changes, perforation, or peritoneal implants, consistent with appendiceal mucocele (Figures 2, 3) [4]. CT imaging is considered the diagnostic modality of choice for appendiceal mucoceles and typically demonstrates a cystic, tubular structure arising from the cecum. Size, wall thickness, and associated findings may help stratify malignant potential [4].

**Figure 2:**
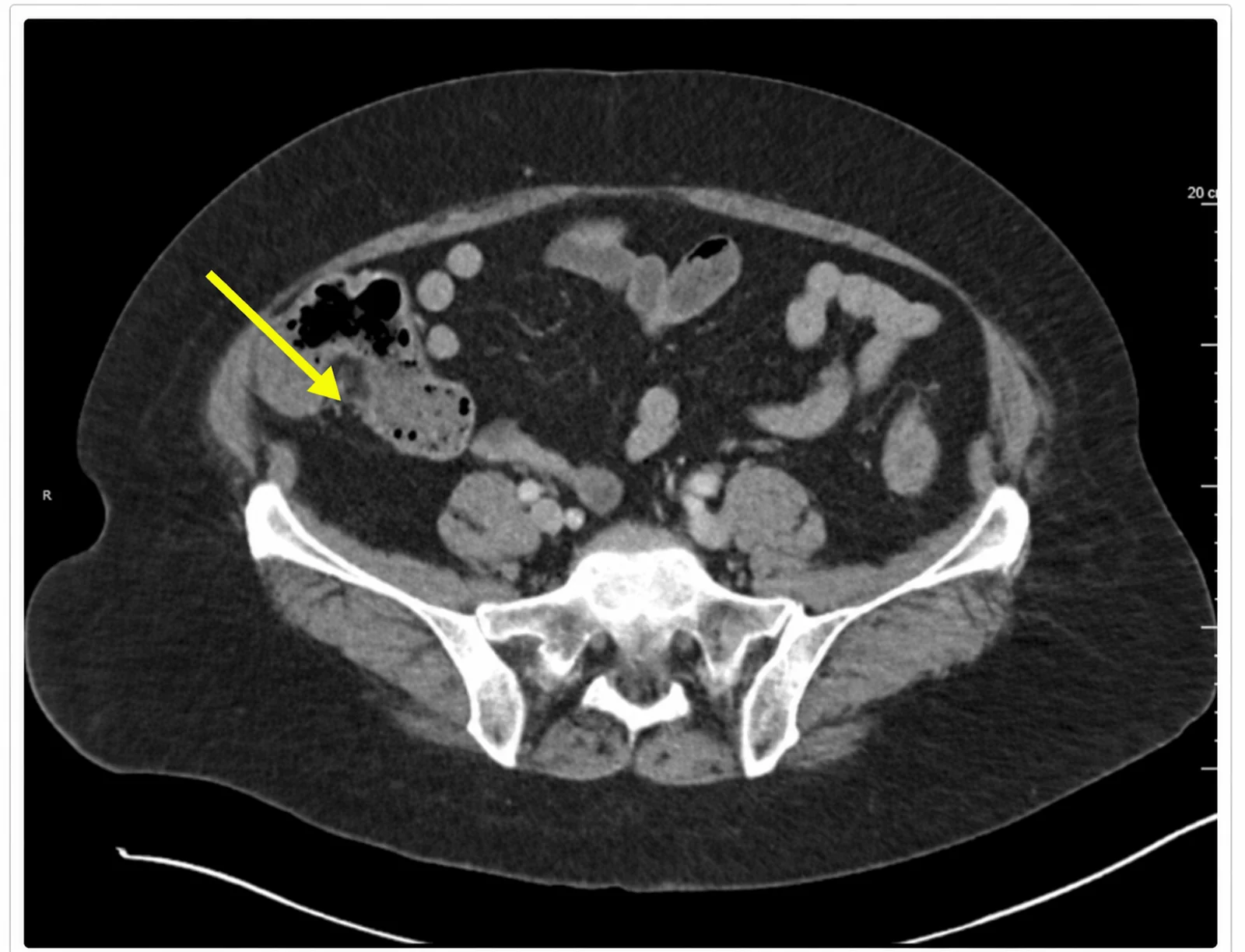
Colonoscopy showing dome-shaped protrusion of the appendiceal orifice with intact overlying mucosa

**Figure 3:**
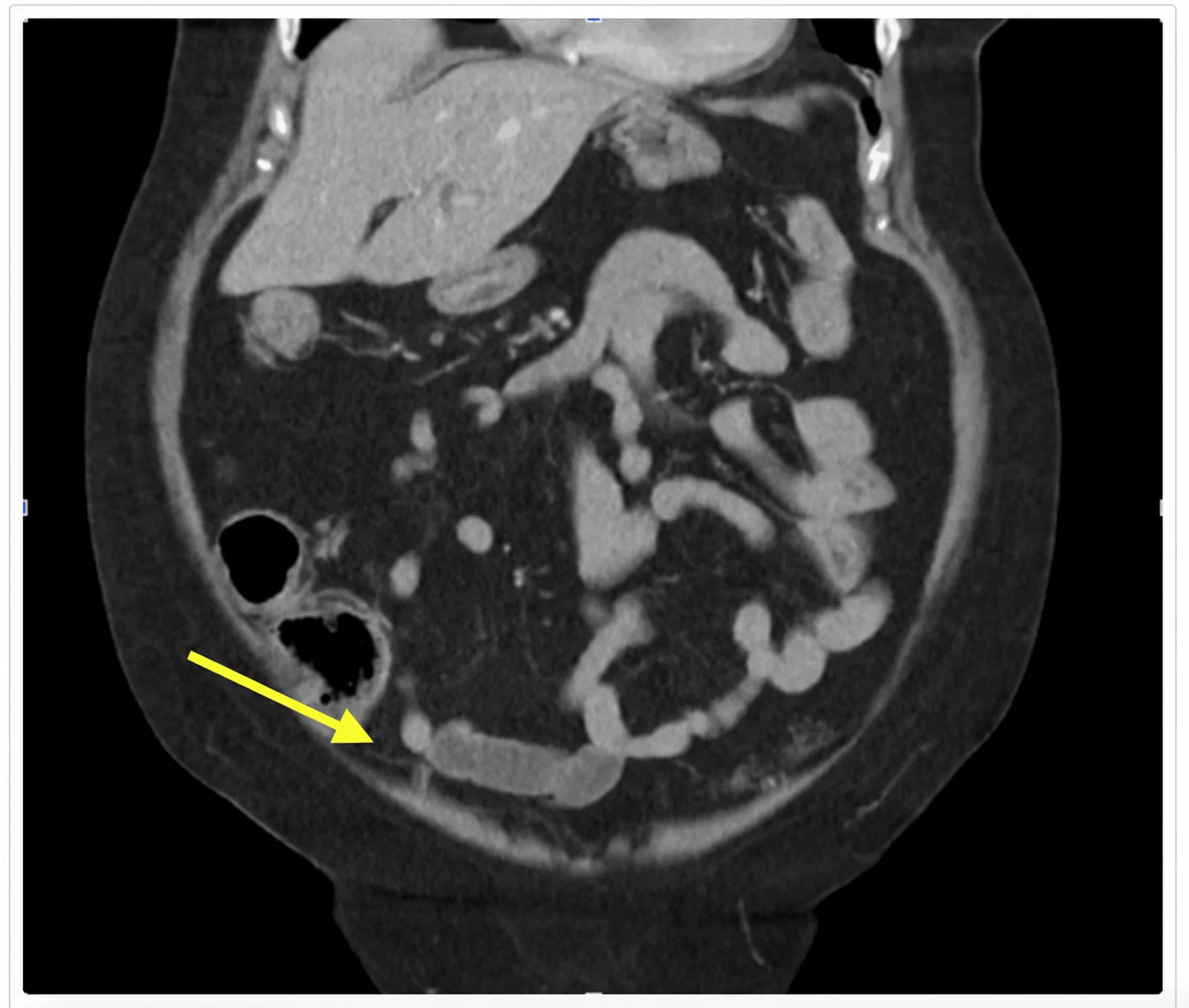
Contrast-enhanced CT showing a dilated, fluid-filled appendix consistent with an appendiceal mucocele

The patient was referred to general surgery and underwent elective laparoscopic appendectomy. The appendix was removed intact without intraoperative rupture or mucin spillage, and no peritoneal implants or extra-appendiceal mucin was identified. Intraoperative frozen-section assessment of the appendiceal margin was negative for neoplastic involvement; therefore, no additional resection, including cecal cuff excision or right hemicolectomy, was performed.

Final histopathologic examination demonstrated luminal dilation by abundant acellular mucin lined by low-grade mucinous epithelium without infiltrative invasion, confirming a low-grade appendiceal mucinous neoplasm (LAMN) confined to the appendix (Figures 4, 5). The final surgical margin was negative, and no extra-appendiceal mucin was identified. The patient recovered uneventfully. Because the lesion was confined to the appendix, completely resected, and unassociated with perforation or extra-appendiceal mucin, no additional surgery or systemic therapy was indicated, and the treating team did not elect dedicated oncologic surveillance [6,7]. Routine postoperative care and age-appropriate colorectal cancer screening were recommended. LAMNs represent a common pathologic diagnosis among mucoceles and carry malignant potential if rupture occurs, supporting definitive surgical management even in asymptomatic patients [5,8].

**Figure 4:**
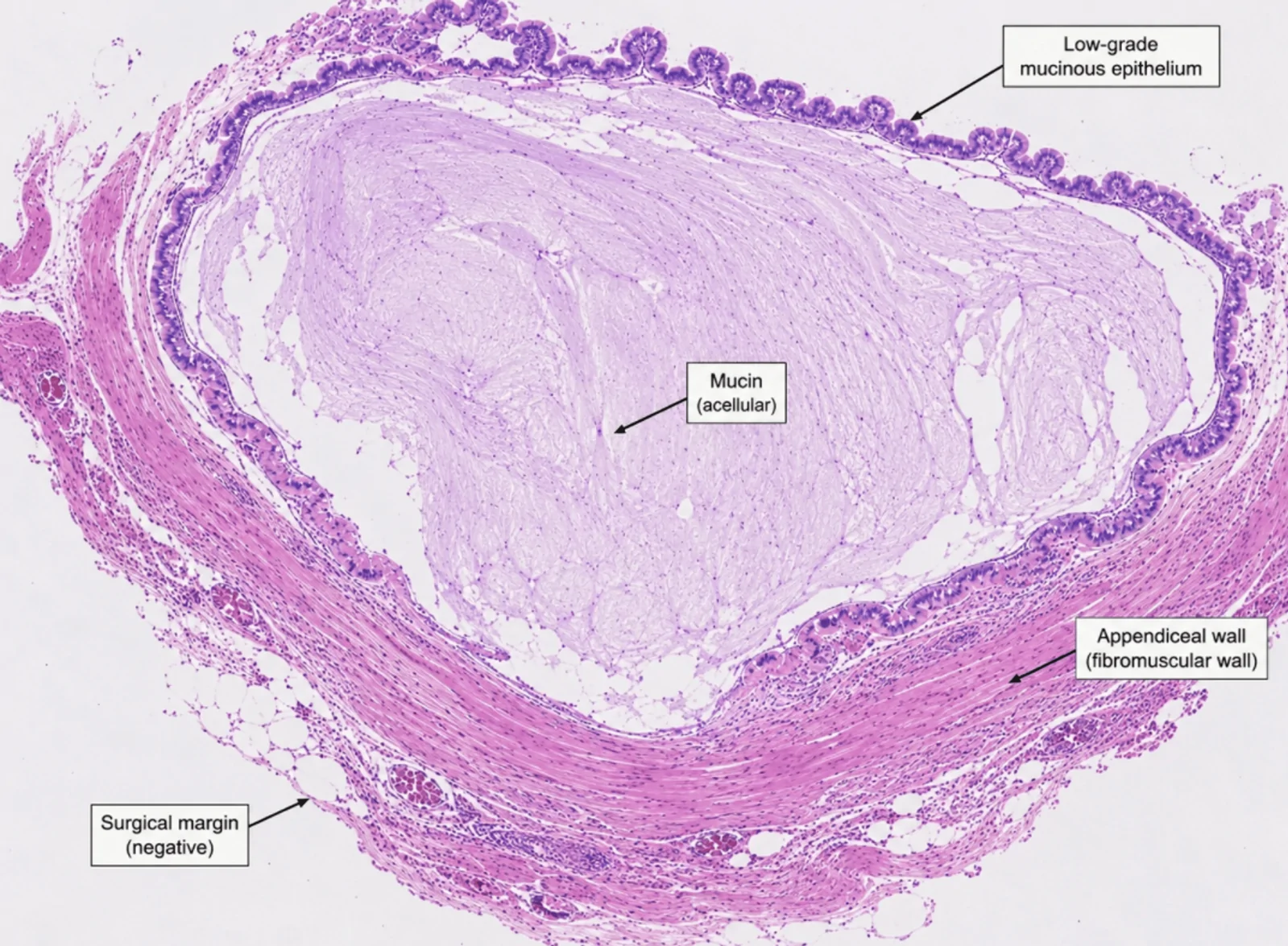
H&E-stained section of the resected appendix showing a low-grade appendiceal mucinous neoplasm with luminal expansion by acellular mucin lined by low-grade mucinous epithelium. The surgical margin is negative (original magnification, ×20).

**Figure 5:**
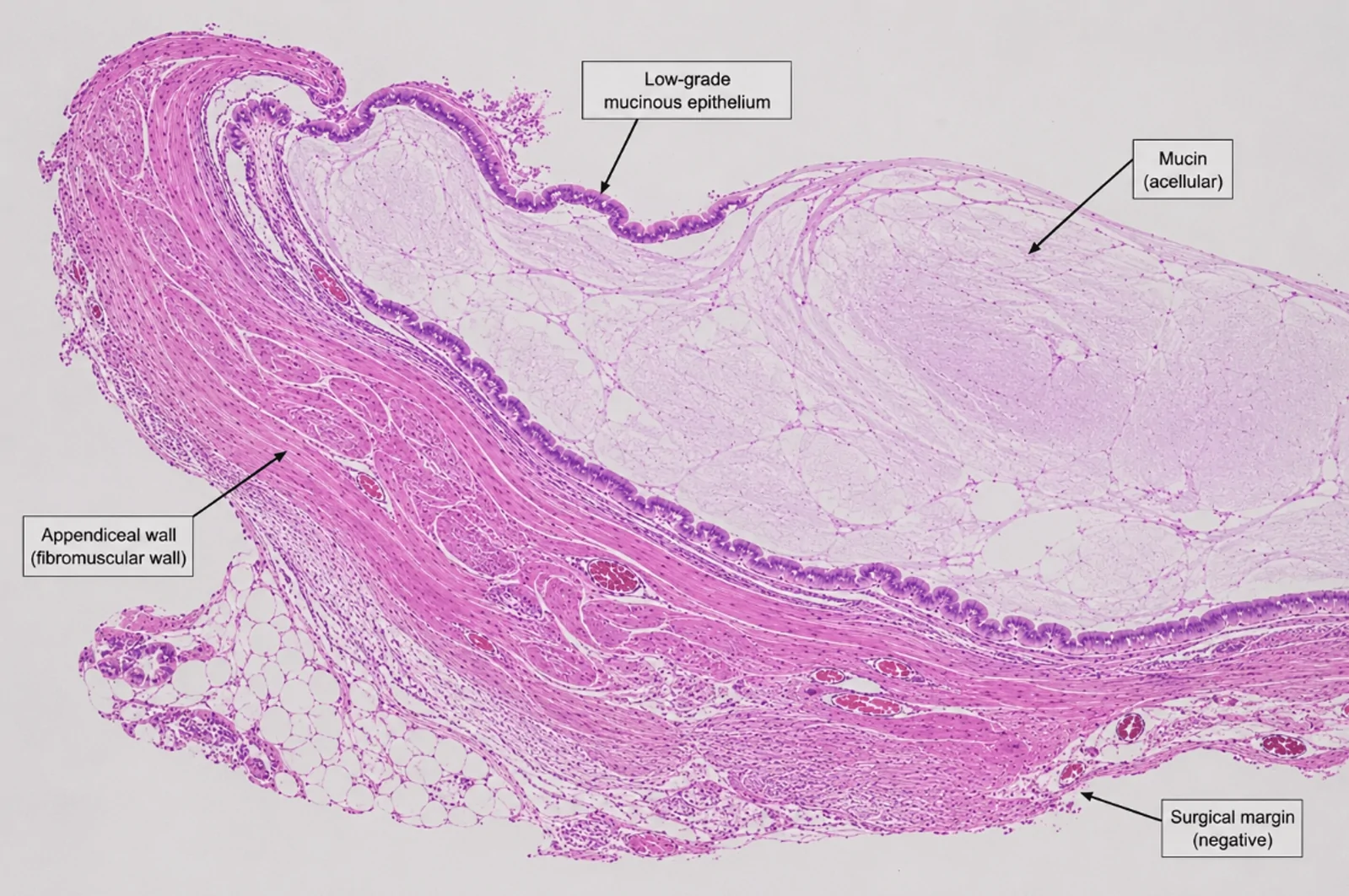
H&E-stained section highlighting the villous low-grade mucinous epithelial lining overlying abundant acellular intraluminal mucin without infiltrative invasion, characteristic of low-grade appendiceal mucinous neoplasm (original magnification, ×20).

## Discussion

This case illustrates why the appendiceal orifice deserves deliberate inspection even during an otherwise routine screening colonoscopy. Appendiceal mucoceles and mucinous neoplasms do not have a specific symptom complex, and patients may report vague abdominal symptoms, present with appendicitis, or remain entirely asymptomatic. In this patient, the only clue was a smooth, dome-shaped bulge centered on the appendiceal opening. Recognition was particularly notable in an asymptomatic man because earlier endoscopic series have reported a female predominance [4]. The case’s rarity lies not in a pathognomonic symptom pattern, but in incidental endoscopic detection before rupture or peritoneal spread.

The macroscopic appearance should determine the immediate endoscopic response. A smooth subepithelial cecal mound, central appendiceal opening, or mucin extrusion should raise suspicion for an appendiceal mucinous lesion. Biopsy is generally low yield because the visible mucosa may be normal, and biopsy or vigorous manipulation may disrupt the lesion. Accordingly, when a mucocele is suspected, the safer next step is cross-sectional imaging rather than tissue sampling [4,5]. This distinction is important: the recommendation applies to a suspected appendiceal mucocele or subepithelial mucinous process and does not prohibit a biopsy of every mucin-containing colorectal lesion.

CT helps confirm appendiceal origin, characterize mural and luminal features, and assess for perforation, extra-appendiceal mucin, and peritoneal disease. Imaging findings associated with appendiceal mucinous neoplasms can include focal appendiceal dilatation greater than 2 cm, curvilinear calcification, wall irregularity, and absence of periappendiceal fat stranding [6,7]. The 2.2-cm thin-walled, fluid-filled appendix in this patient, without inflammatory change or peritoneal implant, supported a localized process and enabled planned resection with attention to avoiding rupture.

Definitive management is directed by operative findings, final histologic classification, margin status, perforation, and the presence and cellularity of extra-appendiceal mucin. Current consensus recommends appendectomy to a negative margin when an appendiceal mucinous neoplasm is localized, and no gross peritoneal disease is present; more extensive treatment and surveillance are reserved for selected adverse pathologic or peritoneal findings [7,8]. In this case, appendectomy alone was appropriate because the LAMN was confined to the appendix, the margin was negative, and neither perforation nor extra-appendiceal mucin was identified.

Because appendiceal tumors are uncommon and classification directly influences management, care should be coordinated through a multidisciplinary team. Current consensus recommends tumor-board discussion and expert pathology review for appendiceal mucinous neoplasms [7]. Depending on disease extent, the team may include a colorectal or surgical oncologist, radiologist, pathologist familiar with appendiceal lesions, and gastrointestinal medical oncologist. Even when appendectomy alone is sufficient, multidisciplinary review can confirm pathologic classification, resection adequacy, and the need for surveillance.

## Conclusions

A subtle appendiceal-orifice bulge in an asymptomatic patient can be the first sign of a clinically consequential appendiceal mucinous neoplasm. Endoscopists should deliberately inspect the appendiceal orifice, recognize a smooth subepithelial mound or volcano-type configuration, and avoid biopsy or forceful manipulation when a mucocele is suspected. The appropriate next steps are cross-sectional imaging and timely surgical evaluation, followed by expert pathology review and multidisciplinary discussion. This sequence can permit complete resection before rupture and reduce the risk of pseudomyxoma peritonei.
